# Supplementary figures and images for: The curious case of the Dana platypus and what it can teach us about how lead shotgun pellets behave in fluid preserved museum specimens and may limit their scientific value
Source: PLoS One. 2024 Oct 18;19(10):e0309845. doi: 10.1371/journal.pone.0309845 (PMC11488718; doi:10.1371/journal.pone.0309845)

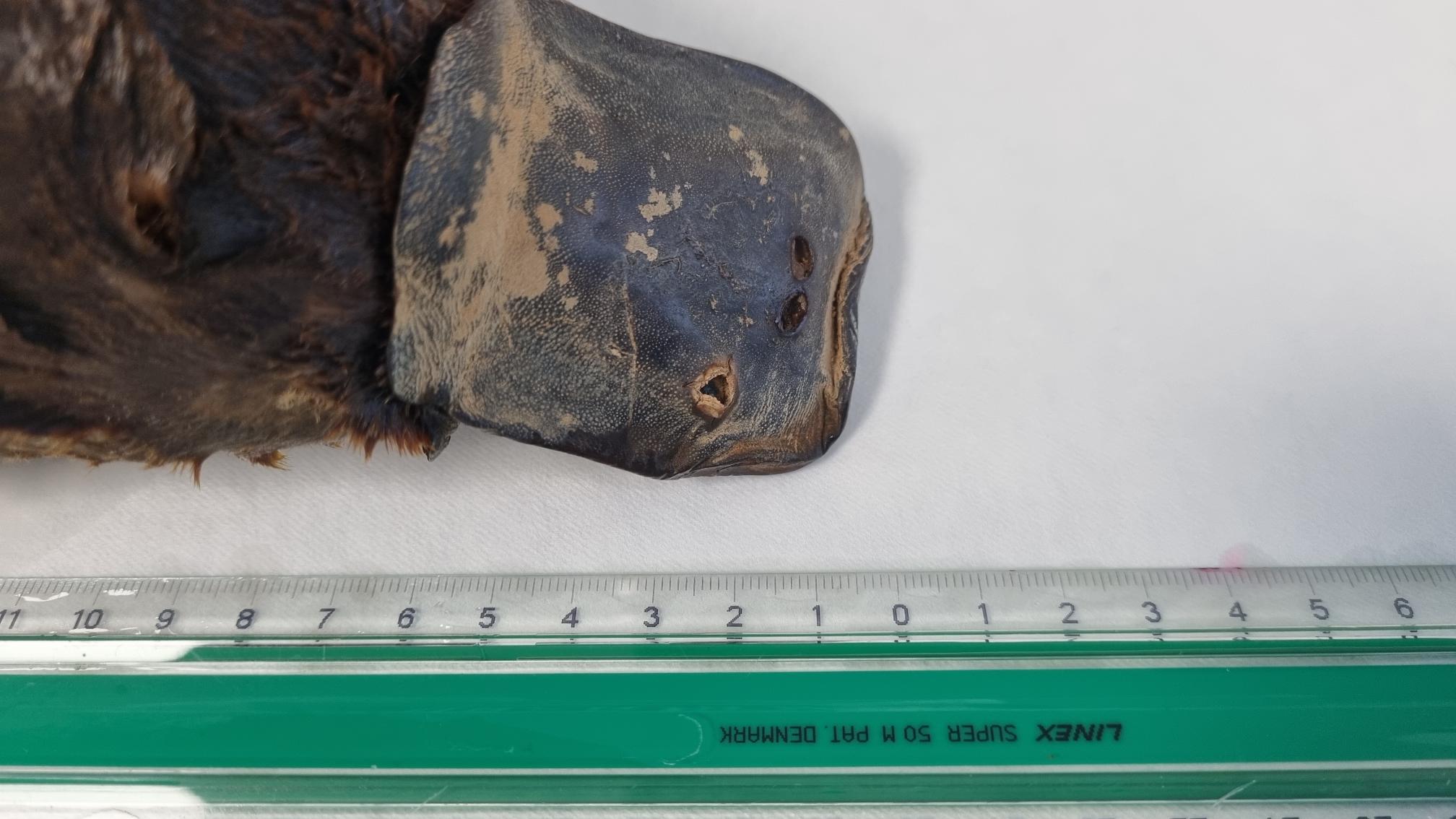

Supplement: S1 File — This material was originally presented to highly trained research and zoo veterinarians (authors CJAW, DS, AKOA and MFB) to form an opinion on what disease the hyperintense nodules in the Dana platypus could originate from. (ZIP) [file pone.0309845.s001.zip › 20230110_103414.jpg]

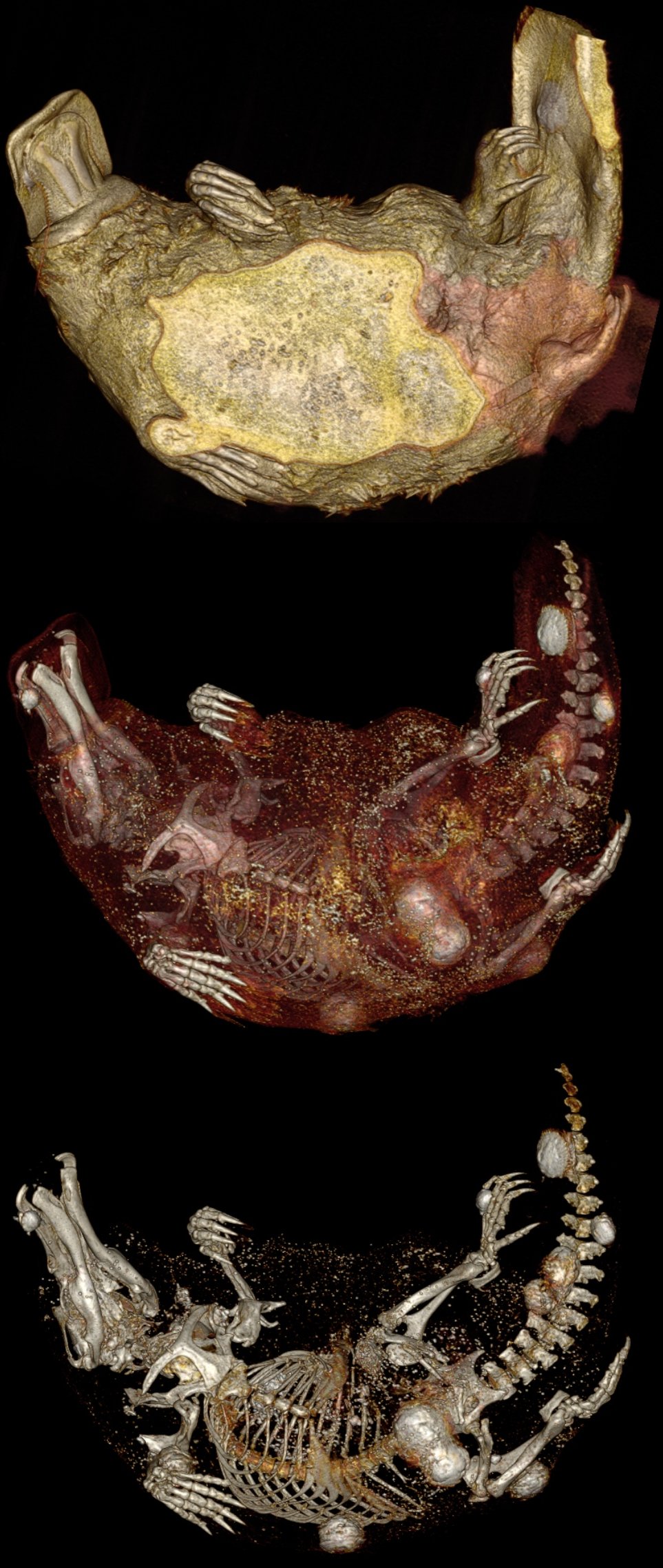

Supplement: S1 File — This material was originally presented to highly trained research and zoo veterinarians (authors CJAW, DS, AKOA and MFB) to form an opinion on what disease the hyperintense nodules in the Dana platypus could originate from. (ZIP) [file pone.0309845.s001.zip › Oana_01_CT_Ventral view_Combined.jpg]

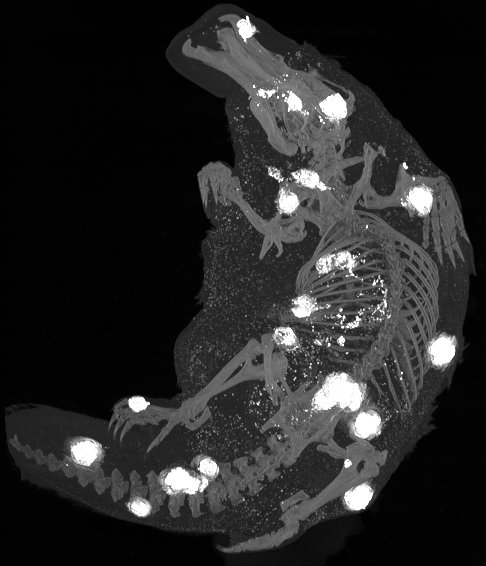

Supplement: S1 File — This material was originally presented to highly trained research and zoo veterinarians (authors CJAW, DS, AKOA and MFB) to form an opinion on what disease the hyperintense nodules in the Dana platypus could originate from. (ZIP) [file pone.0309845.s001.zip › Oana_01_MIP.jpg]
