# Supplementary material for: The curious case of the Dana platypus and what it can teach us about how lead shotgun pellets behave in fluid preserved museum specimens and may limit their scientific value
Source: PLoS One. 2024 Oct 18;19(10):e0309845. doi: 10.1371/journal.pone.0309845 (PMC11488718; doi:10.1371/journal.pone.0309845)
Supplement: Attachment — Submitted filename: Lauridsen et al_Rebuttal letter.docx [file pone.0309845.s003.docx]

First, we would like to express our gratitude to the editor and the two reviewers for considering our manuscript and providing constructive suggestions for improvements of it. In the following, we have addressed all raised points with a response (in red) and with reference to line number (l.) when appropriate in the manuscript with track-changes enabled. Reviewer 1 provided his/her comments in a PDF-version of the manuscript. We have included our response to these below.

PLOS ONE Decision: Revision required [PONE-D-24-21732] - [EMID:13078e117c813891]

em.pone.0.8d588e.6f282465@editorialmanager.com

på vegne af

PLOS ONE<em@editorialmanager.com>

Henrik Lauridsen

PONE-D-24-21732

The curious case of the Dana platypus and what it can teach us about how lead shotgun pellets behave in fluid preserved museum specimens and may limit their scientific value

PLOS ONE

Dear Dr. Lauridsen,

Thank you for submitting your manuscript to PLOS ONE. After careful consideration, we feel that it has merit but does not fully meet PLOS ONE’s publication criteria as it currently stands. Therefore, we invite you to submit a revised version of the manuscript that addresses the points raised during the review process.

Please submit your revised manuscript by Sep 26 2024 11:59PM. If you will need more time than this to complete your revisions, please reply to this message or contact the journal office at [plosone@plos.org](mailto:plosone@plos.org). When you're ready to submit your revision, log on to https://www.editorialmanager.com/pone/ and select the 'Submissions Needing Revision' folder to locate your manuscript file.

Please include the following items when submitting your revised manuscript:

- A rebuttal letter that responds to each point raised by the academic editor and reviewer(s). You should upload this letter as a separate file labeled 'Response to Reviewers'.
- A marked-up copy of your manuscript that highlights changes made to the original version. You should upload this as a separate file labeled 'Revised Manuscript with Track Changes'.
- An unmarked version of your revised paper without tracked changes. You should upload this as a separate file labeled 'Manuscript'.

If you would like to make changes to your financial disclosure, please include your updated statement in your cover letter. Guidelines for resubmitting your figure files are available below the reviewer comments at the end of this letter.

If applicable, we recommend that you deposit your laboratory protocols in protocols.io to enhance the reproducibility of your results. Protocols.io assigns your protocol its own identifier (DOI) so that it can be cited independently in the future. For instructions see: [https://journals.plos.org/plosone/s/submission-guidelines#loc-laboratory-protocols](https://eur01.safelinks.protection.outlook.com/?url=https%3A%2F%2Fc05y1x9s.r.us-east-2.awstrack.me%2FL0%2Fhttps%3A%252F%252Fjournals.plos.org%252Fplosone%252Fs%252Fsubmission-guidelines%2523loc-laboratory-protocols%2F1%2F010f019145cde021-d461bd55-b08d-46e6-9170-7096f223b256-000000%2FA-SJPh854aItMGswCO2gau_4pGY%3D171&data=05%7C02%7Chenrik%40clin.au.dk%7C092255ac0d0b476baef008dcbaacb2d9%7C61fd1d36fecb47cab7d7d0df0370a198%7C1%7C0%7C638590499485557463%7CUnknown%7CTWFpbGZsb3d8eyJWIjoiMC4wLjAwMDAiLCJQIjoiV2luMzIiLCJBTiI6Ik1haWwiLCJXVCI6Mn0%3D%7C0%7C%7C%7C&sdata=0AKSps%2BDRTCZ7%2BZVaP%2BlPKeSugkrVxyJ%2Bf6KpGt67jU%3D&reserved=0). Additionally, PLOS ONE offers an option for publishing peer-reviewed Lab Protocol articles, which describe protocols hosted on protocols.io. Read more information on sharing protocols at [https://plos.org/protocols?utm_medium=editorial-email&utm_source=authorletters&utm_campaign=protocols](https://eur01.safelinks.protection.outlook.com/?url=https%3A%2F%2Fc05y1x9s.r.us-east-2.awstrack.me%2FL0%2Fhttps%3A%252F%252Fplos.org%252Fprotocols%253Futm_medium%3Deditorial-email%2526utm_source%3Dauthorletters%2526utm_campaign%3Dprotocols%2F1%2F010f019145cde021-d461bd55-b08d-46e6-9170-7096f223b256-000000%2FRsV7urG8jR1CH7lHLLV88ZIXvDQ%3D171&data=05%7C02%7Chenrik%40clin.au.dk%7C092255ac0d0b476baef008dcbaacb2d9%7C61fd1d36fecb47cab7d7d0df0370a198%7C1%7C0%7C638590499485570634%7CUnknown%7CTWFpbGZsb3d8eyJWIjoiMC4wLjAwMDAiLCJQIjoiV2luMzIiLCJBTiI6Ik1haWwiLCJXVCI6Mn0%3D%7C0%7C%7C%7C&sdata=3Wj%2BoPnCMNVFAAdF0FLCNtSqgE4Q7PFnSX%2FLUspN4SU%3D&reserved=0).

We look forward to receiving your revised manuscript.

Kind regards,

Gianniantonio Domina, Ph.D.
Academic Editor
PLOS ONE

Journal Requirements:

1. When submitting your revision, we need you to address these additional requirements.

Please ensure that your manuscript meets PLOS ONE's style requirements, including those for file naming. The PLOS ONE style templates can be found at 
<https://journals.plos.org/plosone/s/file?id=wjVg/PLOSOne_formatting_sample_main_body.pdf> and 
<https://journals.plos.org/plosone/s/file?id=ba62/PLOSOne_formatting_sample_title_authors_affiliations.pdf>

We have adjusted the manuscript and believe it now meets PLOS ONE’s style requirements including changing to the Vancouver style of referencing.

2. Thank you for stating the following financial disclosure: "HL is supported by the Carlsberg Foundation (grant# CF21-0605) and PRM is supported by the Carlsberg Foundation (grant# CF21-0435)."

Please state what role the funders took in the study.  If the funders had no role, please state: "The funders had no role in study design, data collection and analysis, decision to publish, or preparation of the manuscript."

If this statement is not correct you must amend it as needed.

Please include this amended Role of Funder statement in your cover letter; we will change the online submission form on your behalf.

We can confirm that the funders had no role in study design, data collection and analysis, decision to publish, or preparation of the manuscript. This sentence has been added to the Funding section (l. 766-767).

3. Thank you for stating the following in the Competing Interests section: "I have read the journal's policy and the author Michiel Krols of this manuscript has the following competing interests: Employed at TESCAN XRE, a manufacture of one of the micro-CT systems used in the study (UniTOM XL Spectral)."

Please confirm that this does not alter your adherence to all PLOS ONE policies on sharing data and materials, by including the following statement: ""This does not alter our adherence to  PLOS ONE policies on sharing data and materials.” (as detailed online in our guide for authors <http://journals.plos.org/plosone/s/competing-interests>).  If there are restrictions on sharing of data and/or materials, please state these. Please note that we cannot proceed with consideration of your article until this information has been declared.

Please include your updated Competing Interests statement in your cover letter; we will change the online submission form on your behalf.

We can confirm that Michiel Krols competing interest does not alter our adherence to PLOS ONE policies on sharing data and materials This sentence has been added to the Competing Interests section (l. 761-762).

4. Please include captions for your Supporting Information files at the end of your manuscript, and update any in-text citations to match accordingly. Please see our Supporting Information guidelines for more information: <http://journals.plos.org/plosone/s/supporting-information>.

We have renamed our Supplementary material section in the end of the manuscript to Supporting information (l. 1077) and have updated the in-text citations.

5. Please review your reference list to ensure that it is complete and correct. If you have cited papers that have been retracted, please include the rationale for doing so in the manuscript text, or remove these references and replace them with relevant current references. Any changes to the reference list should be mentioned in the rebuttal letter that accompanies your revised manuscript. If you need to cite a retracted article, indicate the article’s retracted status in the References list and also include a citation and full reference for the retraction notice.

We do not believe that we cite retracted papers and have not made any changes to the reference list except that we have changed it to the Vancouver style of referencing.

Additional Editor Comments:

Dear Dr. Lauridsen,

the reviewers suggested that your manuscript deserves to be accepted for publication after minor, formal review.
The argument deserves to be published in Plos ONE, the methdology used is suitable with your aims.The form is good.
Please, follow the suggestions of reviewer one to prepare your improved MS.

Best Wishes
Gianniantonio Domina

Reviewers' comments:

Reviewer's Responses to Questions

**Comments to the Author**

1. Is the manuscript technically sound, and do the data support the conclusions?

The manuscript must describe a technically sound piece of scientific research with data that supports the conclusions. Experiments must have been conducted rigorously, with appropriate controls, replication, and sample sizes. The conclusions must be drawn appropriately based on the data presented.

Reviewer #1: Yes

Reviewer #2: Yes

2. Has the statistical analysis been performed appropriately and rigorously?

Reviewer #1: Yes

Reviewer #2: Yes

3. Have the authors made all data underlying the findings in their manuscript fully available?

The [PLOS Data policy](https://eur01.safelinks.protection.outlook.com/?url=http%3A%2F%2Fc05y1x9s.r.us-east-2.awstrack.me%2FL0%2Fhttp%3A%252F%252Fwww.plosone.org%252Fstatic%252Fpolicies.action%2523sharing%2F1%2F010f019145cde021-d461bd55-b08d-46e6-9170-7096f223b256-000000%2F5SpdDWDuGfUU29uGiC-zyuvL5jM%3D171&data=05%7C02%7Chenrik%40clin.au.dk%7C092255ac0d0b476baef008dcbaacb2d9%7C61fd1d36fecb47cab7d7d0df0370a198%7C1%7C0%7C638590499485578680%7CUnknown%7CTWFpbGZsb3d8eyJWIjoiMC4wLjAwMDAiLCJQIjoiV2luMzIiLCJBTiI6Ik1haWwiLCJXVCI6Mn0%3D%7C0%7C%7C%7C&sdata=asr1cOdx2R9a8muqQJbV7GAeXpTfrL%2FVc6cD%2FK5DyDE%3D&reserved=0) requires authors to make all data underlying the findings described in their manuscript fully available without restriction, with rare exception (please refer to the Data Availability Statement in the manuscript PDF file). The data should be provided as part of the manuscript or its supporting information, or deposited to a public repository. For example, in addition to summary statistics, the data points behind means, medians and variance measures should be available. If there are restrictions on publicly sharing data—e.g. participant privacy or use of data from a third party—those must be specified.

Reviewer #1: Yes

Reviewer #2: Yes

4. Is the manuscript presented in an intelligible fashion and written in standard English?

PLOS ONE does not copyedit accepted manuscripts, so the language in submitted articles must be clear, correct, and unambiguous. Any typographical or grammatical errors should be corrected at revision, so please note any specific errors here.

Reviewer #1: Yes

Reviewer #2: Yes

5. Review Comments to the Author

Please use the space provided to explain your answers to the questions above. You may also include additional comments for the author, including concerns about dual publication, research ethics, or publication ethics. (Please upload your review as an attachment if it exceeds 20,000 characters)

Reviewer #1: To the authors:
1/ The manuscript is technically sound, and the offered data support the conclusions
2/ The statistical analysis have been performed appropriately and rigorously
3/ The authors provide all data in the text or in the supplementary data to suport the findings in their manuscript
4/ The English is ok
Comments:
a/ Congratulations on the focus on spirit Natural History Collections.
b/ Just some minor corrections, including in the references section

The reviewer suggests including the scientific name of platypus (*Ornithorhynchus anatinus*) in the title. We have opted not to do this, because the title is not pointing at the species, but in fact a specific individual (the Dana platypus). Instead, in addition to the full species name (including authority) upon first mention of the species in the introduction (l. 85), we have now also inserted the scientific name in the Abstract and in the Keywords sections to increase chances of hits when searching for the species in search engines.

A reference for the flame test has been inserted (l. 225)

Order of references has been updated when changing to the Vancouver style of referencing.

Reviewer #2: Very useful, interesting, and well written.
Since fornaldehyde is a gas at room temperature the expression 4% v/v formaldehyde in misleading. It should be replaced by 3.7% w/v formaldehyde or approximately 4% formaldehyde or 10% v/v formalin (formalin is 37% w/v formadehyde). See: Finkelde, I. and R.R. Waller. 2020. Comparing Methods of Determining Formalin Concentration in Fluid Preservatives. Collection Forum 34 (1): 32–52. <https://doi.org/10.14351/0831-4985-34.1.32>

We completely agree with the reviewer on this point that 4% v/v = 3.7% w/v formaldehyde. But we respectfully disagree that the use of v/v instead of w/v is misleading. The use of the term 4% v/v formaldehyde solution is widespread and describes exactly the same solution with one digit less than 3.7% w/v. So in order to conserve space (in particularly in figures) we prefer to keep 4% v/v formaldehyde.

I expect the most likely lead corrosion product would be lead phosphate. It is unfortunate that X-ray diffraction analysis of the corrosion products could not have been done. Unfortunate also that more sensitive methods of preservative fluid analysis, such as atomic absorption analysis were not employed.

We agree with the reviewer that it would have been of interest to conduct atomic absorption analysis, but unfortunately this was not available to us within the budget of this study. The reviewer may be correct that the corrosion products (or parts of it) may be lead phosphate and we have added this suggestion to the Discussion (l. 650).

These are minor points and overall the manuscript is very good and worthy of publication.

6. PLOS authors have the option to publish the peer review history of their article ([what does this mean?](https://eur01.safelinks.protection.outlook.com/?url=https%3A%2F%2Fc05y1x9s.r.us-east-2.awstrack.me%2FL0%2Fhttps%3A%252F%252Fjournals.plos.org%252Fplosone%252Fs%252Feditorial-and-peer-review-process%2523loc-peer-review-history%2F1%2F010f019145cde021-d461bd55-b08d-46e6-9170-7096f223b256-000000%2FfaCkWiih2cqCXCL71_MrFNQ78Jw%3D171&data=05%7C02%7Chenrik%40clin.au.dk%7C092255ac0d0b476baef008dcbaacb2d9%7C61fd1d36fecb47cab7d7d0df0370a198%7C1%7C0%7C638590499485586419%7CUnknown%7CTWFpbGZsb3d8eyJWIjoiMC4wLjAwMDAiLCJQIjoiV2luMzIiLCJBTiI6Ik1haWwiLCJXVCI6Mn0%3D%7C0%7C%7C%7C&sdata=tuLt6FzKF2o432GBgbiqPmstQCcNTEVS7UWlnONuTH0%3D&reserved=0)). If published, this will include your full peer review and any attached files.

If you choose “no”, your identity will remain anonymous but your review may still be made public.

**Do you want your identity to be public for this peer review?** For information about this choice, including consent withdrawal, please see our [Privacy Policy](https://eur01.safelinks.protection.outlook.com/?url=https%3A%2F%2Fc05y1x9s.r.us-east-2.awstrack.me%2FL0%2Fhttps%3A%252F%252Fwww.plos.org%252Fprivacy-policy%2F1%2F010f019145cde021-d461bd55-b08d-46e6-9170-7096f223b256-000000%2FdTexq_yoXvGTRe2YMDREq0dmqnE%3D171&data=05%7C02%7Chenrik%40clin.au.dk%7C092255ac0d0b476baef008dcbaacb2d9%7C61fd1d36fecb47cab7d7d0df0370a198%7C1%7C0%7C638590499485594051%7CUnknown%7CTWFpbGZsb3d8eyJWIjoiMC4wLjAwMDAiLCJQIjoiV2luMzIiLCJBTiI6Ik1haWwiLCJXVCI6Mn0%3D%7C0%7C%7C%7C&sdata=8Mpe92IFXrXRXW9lVJXUx6O3BZ7BIPbGbGSb27guF8E%3D&reserved=0).

Reviewer #1: No

Reviewer #2: **Yes:** Robert Waller
